# Clinical Outcomes of US Adults Hospitalized for COVID-19 and Influenza in the Respiratory Virus Hospitalization Surveillance Network, October 2021–September 2022

**DOI:** 10.1093/ofid/ofad702

**Published:** 2023-12-30

**Authors:** Noah Kojima, Christopher A Taylor, Mark W Tenforde, Dawud Ujamaa, Alissa O’Halloran, Kadam Patel, Shua J Chai, Pam Daily Kirley, Nisha B Alden, Breanna Kawasaki, James Meek, Kimberly Yousey-Hindes, Evan J Anderson, Kyle P Openo, Libby Reeg, Val Tellez Nunez, Ruth Lynfield, Kathryn Como-Sabetti, Susan L Ropp, Yomei P Shaw, Nancy L Spina, Grant Barney, Sophrena Bushey, Kevin Popham, Nancy E Moran, Eli Shiltz, Melissa Sutton, Nasreen Abdullah, H Keipp Talbot, William Schaffner, Ryan Chatelain, Andrea Price, Shikha Garg, Fiona P Havers, Catherine H Bozio

**Affiliations:** Influenza Division, Centers for Disease Control and Prevention, Atlanta, Georgia, USA; Epidemic Intelligence Service, Centers for Disease Control and Prevention, Atlanta, Georgia, USA; Coronavirus and Other Respiratory Viruses Division, Centers for Disease Control and Prevention, Atlanta, Georgia, USA; Influenza Division, Centers for Disease Control and Prevention, Atlanta, Georgia, USA; Influenza Division, Centers for Disease Control and Prevention, Atlanta, Georgia, USA; Influenza Division, Centers for Disease Control and Prevention, Atlanta, Georgia, USA; Coronavirus and Other Respiratory Viruses Division, Centers for Disease Control and Prevention, Atlanta, Georgia, USA; California Emerging Infections Program, Oakland, California, USA; Centers for Disease Control and Prevention, Atlanta, Georgia, USA; Centers for Disease Control and Prevention, Atlanta, Georgia, USA; Colorado Department of Public Health and Environment, Denver, Colorado, USA; Colorado Department of Public Health and Environment, Denver, Colorado, USA; Connecticut Emerging Infections Program, New Haven, Connecticut, USA; Yale School of Public Health, New Haven, Connecticut, USA; Connecticut Emerging Infections Program, New Haven, Connecticut, USA; School of Medicine, Emory University, Atlanta, Georgia, USA; Georgia Emerging Infections Program, Georgia Department of Public Health, Atlanta, Georgia, USA; Atlanta Veterans Affairs Medical Center, Decatur, Georgia, USA; School of Medicine, Emory University, Atlanta, Georgia, USA; Georgia Emerging Infections Program, Georgia Department of Public Health, Atlanta, Georgia, USA; Atlanta Veterans Affairs Medical Center, Decatur, Georgia, USA; Michigan Department of Health and Human Services, Lansing, Michigan, USA; Michigan Department of Health and Human Services, Lansing, Michigan, USA; Minnesota Department of Health, Saint Paul, Minnesota, USA; Minnesota Department of Health, Saint Paul, Minnesota, USA; New Mexico Department of Health, Albuquerque, New Mexico, USA; New Mexico Department of Health, Albuquerque, New Mexico, USA; New York State Department of Health, Albany, New York, USA; New York State Department of Health, Albany, New York, USA; School of Medicine and Dentistry, University of Rochester, Rochester, New York, USA; School of Medicine and Dentistry, University of Rochester, Rochester, New York, USA; Ohio Department of Health, Columbus, Ohio, USA; Ohio Department of Health, Columbus, Ohio, USA; Public Health Division, Oregon Health Authority, Portland, Oregon, USA; Public Health Division, Oregon Health Authority, Portland, Oregon, USA; Vanderbilt University Medical Center, Nashville, Tennessee, USA; Vanderbilt University Medical Center, Nashville, Tennessee, USA; Salt Lake County Health Department, Salt Lake City, Utah, USA; Salt Lake County Health Department, Salt Lake City, Utah, USA; Influenza Division, Centers for Disease Control and Prevention, Atlanta, Georgia, USA; Coronavirus and Other Respiratory Viruses Division, Centers for Disease Control and Prevention, Atlanta, Georgia, USA; Influenza Division, Centers for Disease Control and Prevention, Atlanta, Georgia, USA

**Keywords:** COVID-19, hospital outcomes, influenza, SARS-CoV-2, severe disease

## Abstract

Severe outcomes were common among adults hospitalized for COVID-19 or influenza, while the percentage of COVID-19 hospitalizations involving critical care decreased from October 2021 to September 2022. During the Omicron BA.5 period, intensive care unit admission frequency was similar for COVID-19 and influenza, although patients with COVID-19 had a higher frequency of in-hospital death.

## BACKGROUND

In the decade prior to the COVID-19 pandemic, seasonal influenza caused an estimated 140 000 to 710 000 hospitalizations and 12 000 to 52 000 deaths in the United States annually [1]. After very low levels of influenza circulation during the first year of the COVID-19 pandemic, circulation increased during the 2021–2022 season, with A(H3N2) viruses predominating [2]. The epidemiology of COVID-19 also changed during the pandemic, with circulation of new variants and increasing population-level immunity [3]. It is therefore important to monitor the changing epidemiology of COVID-19 and influenza over time with clinically relevant metrics of severity. Using a population-based hospitalization surveillance network, we describe patient characteristics and clinical outcomes of adults hospitalized with laboratory-confirmed SARS-CoV-2 or influenza in the United States from 1 October 2021 to 30 September 2022.

## METHODS

The Respiratory Virus Hospitalization Surveillance Network—which includes the Coronavirus Disease 2019–Associated Hospitalization Surveillance Network (COVID-NET) and the Influenza Hospitalization Surveillance Network (FluSurv-NET)—conducts population-based surveillance for laboratory-confirmed COVID-19– and influenza–associated hospitalizations, respectively [4]. During 2021 to 2022, surveillance for COVID-19 and influenza hospitalizations was conducted in selected counties in 14 states. For additional information about COVID-NET or FluSurv-NET, see Supplement 1.

For this analysis, we included adult patients who were hospitalized for COVID-19 (1 October 2021–30 September 2022) or influenza (1 October 2021–30 April 2022) in 12 of the 14 states (Supplemental 1). We classified patients hospitalized for COVID-19 into SARS-CoV-2 variant periods based on the nationally predominant circulating variant at the time of admission [5]. We defined the Delta-predominant period as 1 October to 18 December 2021. The subsequent Omicron-predominant period was divided into 3 subvariant-predominant periods: BA.1 from 19 December 2021 to 19 March 2022, BA.2 from 20 March to 18 June 2022, and BA.5 from 19 June to 30 September 2022. Influenza hospitalizations were analyzed together from 1 October 2021 to 30 April 2022. The Omicron BA.5–predominant period was compared with the 2021–2022 influenza season as it was the most recent subvariant in the surveillance period.

Medical records were abstracted via a standardized case report form to obtain information on demographics, clinical characteristics, and interventions that indicated increased severity and outcomes during hospitalization. *Severity* was defined as high-flow nasal cannula (HFNC), noninvasive positive pressure ventilation (NIPPV), invasive mechanical ventilation (IMV), vasopressors, renal replacement therapy (RRT), or extracorporeal membrane oxygenation (ECMO). *Outcomes* were defined as intensive care unit (ICU) admission and in-hospital death. Data were abstracted for all FluSurv-NET case patients and an age- and site-stratified representative sample of COVID-NET case patients [6].

Patients coinfected with influenza virus and SARS-CoV-2, children (<18 years old), and pregnant individuals were excluded. We further restricted our analysis to sampled adults who were likely admitted for either COVID-19 or influenza (see Supplement 2 for the details on classifying “reason for admission”).

Demographic characteristics and in-hospital outcomes were reported with unweighted counts and weighted percentages to account for COVID-NET sampling. Proportions with select clinical outcomes were compared between COVID-19 cases during the Omicron BA.5 period and influenza cases through chi-square tests with weighted proportions. The Cochran-Armitage test for trend tested whether the frequency of outcomes among COVID-NET cases changed over COVID-19 variant–predominant periods, not accounting for sampling weights [7]. Analyses were performed in aggregate and stratified by age (18–49, 50–64, 65–74, and ≥75 years). *P* < .05 indicated statistical significance and accounted for sampling weights. Analyses were completed with Stata SE version 17.0 (StataCorp) and SAS version 9.4 (SAS Institute).

These activities were reviewed by the Centers for Disease Control and Prevention and conducted consistent with applicable federal law and CDC policy (eg, 45 CFR part 46.102[l][2], 21 CFR part 56; 42 USC §241[d], 5 USC §552a, 44 USC §3501 et seq). Sites participating in COVID-NET and FluSurv-NET obtained human subjects and ethics approvals from their state and local health departments and academic partner institutional review boards as needed.

## RESULTS

A total of 166 040 COVID-19 hospitalizations (1 October 2021–30 September 2022) and 3536 influenza hospitalizations (1 October 2021–30 April 2022) were reported. After application of exclusion criteria, 5777 (3.5%) and 2363 (66.8%) patients remained in the analytic population for COVID-19 and influenza, respectively (Supplementary Figure 1). Among hospitalizations in the Delta-predominant period (n = 1632), in the Omicron BA.5–predominant period (n = 1451), and for influenza (n = 2363), the median ages were 59, 61, and 68 years; 46.5%, 52.3%, and 53.0% were female; and 76.4%, 91.6%, and 90.7% had ≥1 underlying condition, respectively. Among Delta- and Omicron BA.5–predominant period hospitalizations, 29.6% and 70.3% of patients received at least a primary COVID-19 vaccination series; for influenza hospitalizations, 56.2% had received a current season influenza vaccine. Demographic and clinical data stratified by age groups are displayed in Supplementary Table 1.

**Figure 1.**
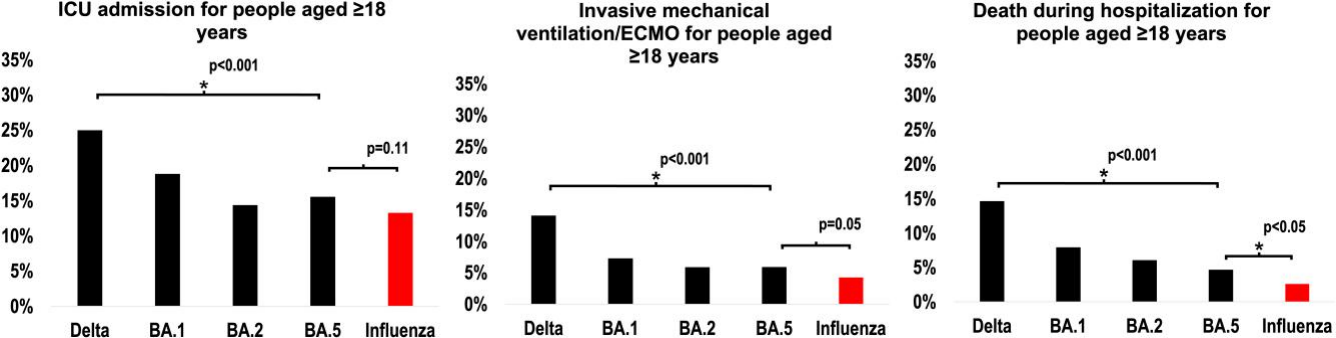
Percentage of adult patients admitted for COVID-19 by variant/subvariant predominance period and percentage of patients admitted for COVID-19 Omicron BA.5 vs influenza (RESP-NET, 2021–2022) for the following outcomes: *A*, ICU admission; *B*, invasive mechanical ventilation/ECMO; *C*, death. *A statistically significant change in trend was assessed with the Cochran-Armitage test. A statistically significant difference between COVID-19 Omicron BA.5 and influenza was assessed through logistic regression. ECMO, extracorporeal membrane oxygenation; ICU, intensive care unit; RESP-NET, Respiratory Virus Hospitalization Surveillance Network.

The percentage of COVID-19 adult patients who were admitted to the ICU, received IMV/ECMO, and experienced in-hospital death decreased from the Delta- to Omicron BA.5–predominant period (all *P* < .05; Figure 1). This decrease was observed in all groups (all *P* < .05), except for ICU admission and IMV/ECMO receipt among those aged 18 to 49 years (Supplementary Figures 2–5).

Regarding clinical outcomes between COVID-19 Omicron BA.5 and influenza hospitalizations, a similar percentage of patients was admitted to the ICU (15.5% vs 13.3%) and received HFNC (7.1% vs 9.5%), NIPPV (11.7% vs 14.4%), and IMV/ECMO (5.9% vs 4.2%, all *P* > .05; Supplementary Table 2). The percentage was higher among those with Omicron BA.5 than influenza for vasopressor use (8.2% vs 4.8%), RRT (5.2% vs 3.4%), and in-hospital death (4.6% vs 2.6%, all *P* < .05). Stratified by age, death during hospitalization was higher only for Omicron BA.5 among those aged 18 to 49 years; for all other age groups, there was no difference in deaths during hospitalization for Omicron BA.5 and influenza (Supplementary Figures 2c, 3c, 4c, and 5c).

## DISCUSSION

In a geographically diverse population-based surveillance network, adults hospitalized for either COVID-19 or influenza during 2021 to 2022 experienced substantial morbidity and mortality. The percentage of hospitalized patients with COVID-19 who experienced severe outcomes such as ICU admission, HFNC, NIPPV, vasopressors, IMV/ECMO, and in-hospital death decreased from the Delta- to Omicron BA.5–predominant period. The percentage of hospitalized patients with COVID-19 admitted to the ICU was similar to that for seasonal influenza, but during the Omicron BA.5 period, the former continued to experience more in-hospital death than adults hospitalized with influenza. The association with increased in-hospital death was strongest among adults aged 18 to 49 years; there was no significant difference in deaths between patients with COVID-19 and influenza among older ages.

The epidemiology of COVID-19 continues to evolve. Our data suggest that from the Delta–predominant period to the Omicron BA.5–predominant period, the severity of patients hospitalized with COVID-19 generally decreased [3]. This is demonstrated by the declining percentage of adult patients hospitalized for COVID-19 during the Omicron BA.5–predominant period who were admitted to the ICU, received an intervention (HFNC, NIPPV, vasopressors, IMV/ECMO), and experienced in-hospital death, similar to analyses conducted in other surveillance systems [3]. This reduced severity is likely multifactorial, including contributions from increased population-level SARS-CoV-2 immunity from vaccination and prior infections, greater availability of effective therapeutics, and updated clinical management strategies [8, 9]. Although there was a decline in the severity of COVID-19 hospitalization during the study period, overall severity during later Omicron sublineage periods (BA.2 and BA.5) was generally similar. Continued monitoring of severity trends is warranted as new variants circulate and population-level immunity changes.

Similar percentages of patients hospitalized for COVID-19 during the BA.5 period and for influenza were admitted to the ICU and received respiratory support, although the percentage of patients receiving vasopressors or RRT and experiencing in-hospital death was greater among COVID-19 cases than influenza cases. Other studies found that when compared with seasonal influenza, COVID-19 was associated with an increased risk of extrapulmonary organ dysfunction and death [10], which is consistent with our findings that a higher percentage of patients with Omicron BA.5 experienced death and receipt of vasopressors and RRT vs seasonal influenza. The percentage of patients hospitalized with COVID-19 who received respiratory support likely decreased in part because (1) infections with the Omicron variant in immunized persons have been associated with lower rates of acute lung injury and systemic inflammation and (2) the percentage of patients who received at least a primary COVID-19 vaccination rose over the surveillance period [3, 11]. Additionally, changes in clinical management strategies over time, including increased use of HFNC and delayed initiation of IMV, might account for lower rates of invasive oxygenation strategies during later periods [12].

This analysis has several limitations. First, clinician-driven testing was used to identify cases for influenza and COVID-19. Facilities implementing universal SARS-CoV-2 testing may have detected COVID-19 more completely than influenza. Second, misclassification of whether an admission was for COVID-19 or influenza could have occurred, although a standardized algorithm was used to reduce misclassification. Third, evolving clinical practice patterns, such as changing hospitalization and ICU admission practices and use of therapeutics, could have contributed to differences in outcomes over time. Fourth, these results may not be nationally representative. Fifth, periods of variant predominance are based on national data and might not reflect regional differences. Sixth, deaths include only in-hospital deaths, and deaths that occurred after hospitalization were not assessed. Seventh, excluded cases are unevenly distributed between COVID-19 and influenza arms, which could bias patient characteristics. Therefore, we stratified our analysis by age in part to understand such differences. Despite differences in the arms, we saw consistent trends within age groups, which provided reassurance in the validity of our overall findings.

## CONCLUSIONS

Despite declines in COVID-19 severity, this analysis demonstrates that COVID-19 and influenza continue to cause severe disease among hospitalized patients. Furthermore, optimizing disease prevention and treatment strategies, such as vaccinations, antiviral medications, and nonpharmaceutical interventions, may attenuate severe disease.

## Supplementary Material

ofad702_Supplementary_Data

## Supplementary Data

Supplementary materials are available at *Open Forum Infectious Diseases* online. Consisting of data provided by the authors to benefit the reader, the posted materials are not copyedited and are the sole responsibility of the authors, so questions or comments should be addressed to the corresponding author.

## Notes

***Acknowledgments***. We acknowledge all personnel and sites in the Respiratory Virus Hospitalization Surveillance Network.

***Patient consent statement***. This study was conducted with surveillance data and does not include factors necessitating patient consent.
