# Supplementary material for: Clinical Outcomes of US Adults Hospitalized for COVID-19 and Influenza in the Respiratory Virus Hospitalization Surveillance Network, October 2021–September 2022
Source: Open Forum Infect Dis. 2023 Dec 30;11(1):ofad702. doi: 10.1093/ofid/ofad702 (PMC10807992; doi:10.1093/ofid/ofad702)
Supplement: ofad702_Supplementary_Data [file ofad702_supplementary_data.zip › RESPNETCOVIDvFluManuscript_11_30_23 OFID Supplement.docx]

Supplement

ACKNOWLEDGEMENTS

CA: Brenna Hall, Gretchen Rothrock, Jeremy Roland, Joelle Nadle, Ashley Coates, Monica Napoles, California Emerging Infections Program

CO: Elizabeth Austin, Isaac Armistead, Diane Garcia, LeAnna Kent, Colorado Department of Public Health and Environment

CT: Ann Basting, Tessa Carter, Maria Correa, Julia Desiato, Sarah Katsandres, Daewi Kim, Hannah Litwak, Carol Lyons, Molly McLaughlin, Julie Plano, Hazhia Sorosindi, Connecticut Emerging Infections Program, Yale School of Public Health;

GA: Emily Fawcett, Annabel Patterson, Taylor Eisenstein, Gracie Chambers: Foundation for Atlanta Veterans Education and Research, Decatur, GA; Georgia Emerging Infections Program, Georgia Department of Public Health, Atlanta, GA Atlanta Veterans Affairs Medical Center, Decatur, GA;

Katelyn Ward, Jana Manning, Asmith Joseph, Allison Roebling, Chandler Surell, Stephanie Lehman, Suzanne Segler, Grayson Kallas, Marina Bruck, Rayna Ceaser, Sabrina Hendrick, Johanna Hernandez, Hope Wilson; Emory University School of Medicine, Atlanta, GA; Georgia Emerging Infections Program, Georgia Department of Public Health, Atlanta, GA. Atlanta Veterans Affairs Medical Center, Decatur, GA

MI: Jim Collins, Shannon Johnson, Justin Henderson, Sue Kim, Chloe Brown, Lauren Leegwater, Anna Falkowski, Alyanna Melicor, Sanchitha Meda, Michigan Department of Health and Human Services;

MN: Erica Bye, Paige D’Heilly, Cynthia Kenyon, Melissa McMahon, Stephanie Meyer, Erica Mumm, Anna Strain, Xiong Wang, Jennifer Zipprich, Minnesota Department of Health;

NM: Daniel M. Sosin, Chad Smelser, Sunshine Martinez, Jasmyn Sanchez, Cory Cline, Melissa Judson, Florent Nkouaga, Mark Montoya, Adrienne Domen, Kelly Plymesser, New Mexico Department of Health; Sarah Lathrop, Kathy M. Angeles, Sarah A. Khanlian, Mayvilynne Poblete, Zachary Landis, Emily Hancock, Yadira Salazar-Sanchez, Nancy Eisenberg, Dominic Rudin, Wickliffe Omondi, Francesca Pacheco, Molly Bleecker, New Mexico Emerging Infections Program; Yassir Talha, Celina Chavez, Jennifer Akpo, Alesia Reed, Murtada Khalifa, CDC Foundation, New Mexico Department of Health;

NYA: Kerianne Engesser, Adam Rowe, Suzanne McGuire, Jemma Rowlands, Bridget Anderson, New York State Department of Health;

NYR: Christina Felsen, Maria Gaitan, Erin Licherdell, Christine Long, Katherine St. George, University of Rochester School of Medicine and Dentistry;

OH: Laurie Billing, Denise Ingabire-Smith, Rebekah Sutter, Ohio Department of Health;

OR: M. Andraya Hendrick, Sam Hawkins, Public Health Division, Oregon Health Authority

TN: Tiffanie Markus, William Schaffner, Katie Dyer, Karen Leib, Terri McMinn, Danielle Ndi, Gail Hughett, Bentley Akoko, Kathy Billings, Anise Elie, Vanderbilt University Medical Center;

UT: Amanda Carter, Andrea George, Andrew Haraghey, Ashley Swain, Ashton Bruno, Courtney H. Sacco, Emma Mendez, Hafsa Zahid, Isabella Reyes, Kristen P. Olsen, Mary Hill, Melanie T. Crossland, Rosie Gonzalez, Holly Staten, Salt Lake County Health Department

Supplemental Table 1. Demographic and clinical characteristics of adults hospitalized for COVID-19 or influenza stratified by age group and COVID-19-predominant period, RESP-NET, 2021-2022

See Excel Sheet Supplemental Table 1.

Abbreviations: IQR=interquartile range

*known (n) only presented for variables with missing data.

**at least a primary COVID-19 vaccination series was defined as those with a positive SARS-CoV-2 test result from a specimen collected ≥14 days after either the second dose of a 2-dose vaccination series or after 1 dose of a single-dose vaccine (i.e., Janssen / Johnson & Johnson). A current season influenza vaccine was defined as one received at least 2 weeks prior to hospitalization. COVID vaccination data were collected on COVID cases and influenza vaccination data were collected on influenza cases.

***counts are unweighted and the percentages are weighted.

Supplemental Table 2. Outcomes of adults hospitalized for COVID-19 or influenza stratified by age group and COVID-19-predominant period, RESP-NET, 2021-2022

See Excel Sheet Supplemental Table 2.

Abbreviations: ICU=intensive care unit; NIPPV=non-invasive positive pressure ventilation; ECMO=extracorporeal membrane oxygenation.

*known (n) only presented for variables with missing data.

**calculated with Rao-Scott Chi Square

***counts are unweighted and the percentages are weighted.

Supplement 1: Notes on RESP-NET surveillance, testing, and case definition.

During the 2021­-2022 season, due to late season influenza activity, FluSurv-NET surveillance was extended through 6/30/2022, though clinical data were only collected on cases admitted through the end of 4/30/2022.

A COVID-NET or FluSurv-NET case was defined as a hospitalized resident of the site’s catchment area, with a positive SARS-CoV-2 (rapid antigen detection or molecular assay) or influenza (rapid antigen detection, molecular assay, direct or indirect immunofluorescence assay, or viral culture) test during hospitalization or within 14 days prior to hospitalization, respectively. SARS-CoV-2 and influenza virus testing were performed at the discretion of healthcare practitioners or according to hospital testing practices. Trained surveillance staff identified all catchment area residents hospitalized with COVID-19 or influenza using laboratory, hospital, and reportable conditions databases.

Supplement 2: Standard Operating Procedure to classify patients likely admitted for either COVID-19 or influenza.

Collection and reclassification of reason for admission

“Reason for admission” was added to the COVID-NET case report form (CRF) in June 2020 and added to the FluSurv-NET case report form (CRF) during the 2021-2022 influenza season to distinguish between patients primarily admitted for COVID-19-related illness versus non-COVID-19-related illness in the COVID-NET dataset and to distinguish between patients admitted for influenza-related illness versus non-influenza-related illness in the FluSurv-NET dataset. There were six mutually exclusive response options to the “Reason for admission” variable which included 1) COVID-19 related illness (for COVID-NET) or Influenza-related illness (for FluSurv-NET); 2) Obstetrics/Labor and delivery admission; 3) Inpatient surgery/procedures for planned procedures; 4) Psychiatric admission needing acute medical care; 5) Trauma; and 6) Other. The response option of “Other” to the “Reason for admission” variable allowed RESP-NET surveillance officers to enter free text to describe a reason for admission different from the other five categories of reason for admission.

A Natural Language Processing (NLP) Machine Learning (ML) algorithm was developed to recategorize the free text entered as “other” reason for admission into one of the existing five reason for admission categories or to leave it categorized as “other”.  To develop the first training dataset for the NLP ML algorithm, general rules were agreed upon to guide any potential reclassification of the reason for admission: 1) recategorizing any exacerbation of an underlying condition (e.g., congestive heart failure, diabetes, or sickle cell anemia) as influenza- or COVID-19-related illness, 2) recategorizing any cardiac or neurologic complication (e.g., myocardial infarction or stroke) as influenza- or COVID-19-related illness, 3) recategorizing any falls, confusion, mental status changes, loss of consciousness, or weakness for adults as influenza- or COVID-19-related illness, 4) considering the presence of respiratory symptoms when recategorizing, 5) taking into consideration indications from surveillance officers that the reason for admission was not likely COVID-19 or influenza-related illness, and 6) leaving acute non-respiratory infection (e.g., abscess, appendicitis, diabetic foot infection, or osteomyelitis) categorized as “other”.  Three clinicians supporting COVID-NET and five clinicians supporting FluSurv-NET then independently reviewed the unique list of other reason for admission free text for influenza and either recategorized the free text as one of the five reasons for admission categories or left the free text categorized as “other”.  The same process was used for COVID-19 admissions, with review by three physicians. Adjudication was done for any discrepancy in categorization between the clinicians to come to a consensus for categorization of the free text.  After the generation of the initial training dataset, the original free text for other reason for admission was used as a test and validation dataset on which the NLP ML algorithm could predict the reason for admission category. The clinicians then reviewed and validated the predicted categorization produced by the NLP ML algorithm, and the new validated categorizations were appended to the training dataset in an iterative process.

The NLP ML algorithm was developed using the Scikit-learn and the Natural Language Toolkit in Python 3.9.13.   The steps in the NLP ML algorithm included: 1) importing the training dataset of free text categorized by the RESP-NET clinicians; 2) text normalizing (transforming the text to all lower characters), tokenizing (splitting phrases and sentences into individual word tokens), and removing punctuation from the free text; 3) defining the NLP ML pipeline for multi-class categorization using Scikit-learn’s Count Vectorizer [1], TfidfTransformer (Term-Frequency inverse document-frequency Transformer) [2], and Stochastic Gradient Descent Classifier [3]; 4) training the NLP ML pipeline on the training dataset; 5) running the training NLP ML pipeline on the test dataset of new other reason for admission free text; and 6) using the FuzzyWuzzy [4] library in Python for fuzzy string matching free text in the test dataset with the same category as free text in the training dataset if there was a match between 90% or more of the free text in both datasets.

Ultimately, the most common free text entries that were recategorized from other reason for admission to COVID-19-related or Influenza-related illness were: abdominal pain, fall, chest pain, altered mental status, seizure, syncope, diabetic ketoacidosis/DKA, weakness, stroke, and cardiac arrest.  The most common free-text entries that were recategorized from other reason for admission to a non-COVID-19 related or non-Influenza-related illness were: chemotherapy (Inpatient surgery/procedures), dialysis (Inpatient surgery/procedures), alcohol withdrawal (Psychiatric admission needing acute medical care), suicidal ideation (Psychiatric admission needing acute medical care), burns (Trauma), appendicitis (Other), and Newborn (Other).

**References**

1. F. Pedregosa et al., "CountVectorizer—Scikit-Learn 1.3.0 Documentation," scikit-learn: machine learning in Python (2020), https://scikit-learn.org/stable/modules/generated/sklearn.feature_extraction.text.CountVectorizer.html
2. F. Pedregosa et al., "TfidfTransformer—Scikit-Learn 1.3.0 Documentation," scikit-learn: machine learning in Python (2020), [https://scikit-learn.org/stable/modules/generated/sklearn.feature_extraction.text.TfidfTransformer.html](about:blank)
3. F. Pedregosa et al., "SGDClassifier—Scikit-Learn 1.3.0 Documentation," scikit-learn: machine learning in Python (2020), [https://scikit-learn.org/stable/modules/generated/sklearn.linear_model.SGDClassifier.html](about:blank)
4. A. Cohen et al., "FuzzyWuzzy: Fuzzy String Matching in Python" (2011), https://github.com/seatgeek/fuzzywuzzy

Supplemental Figure 1. Flow chart of inclusion and exclusion criteria for adults aged ≥18 years and hospitalized with either laboratory-confirmed COVID-19 or influenza from October 1, 2021 through April 30, 2022


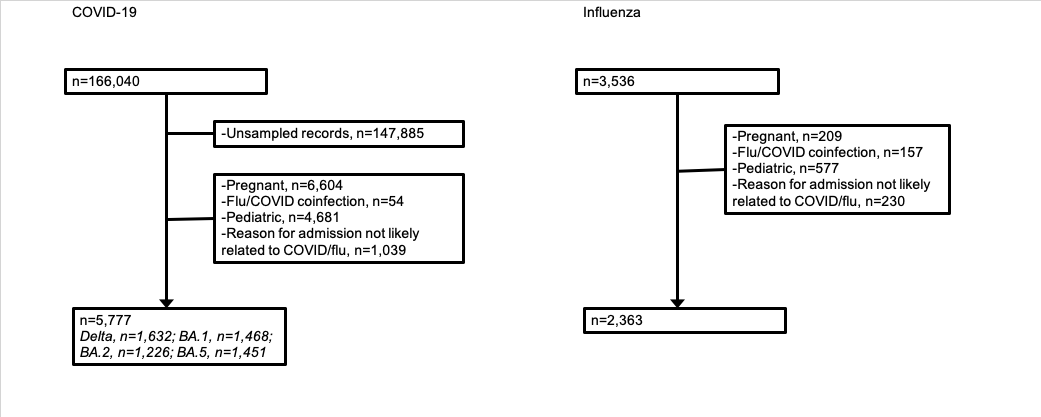


*Data from Iowa and Maryland were excluded from the analysis because Iowa did not contribute clinical data on cases and Maryland had a gap in COVID-19 surveillance for a period during 2021­-2022 season. Patient data from Iowa and Maryland were not included in the n=166,040 for COVID-19 nor n=3,536 for influenza.

**Exclusion criteria was applied to cohort in the order listed in the flow chart.

Supplemental Figure 2 (Left to right). Comparison of percentage of adult patients aged 18-49 years admitted for COVID-19 by COVID-19 variant/subvariant predominance period and a comparison of the percentage of patients admitted for COVID-19 Omicron BA.5 versus influenza, RESP-NET, 2021-2022, for the following outcomes: 2a) ICU admission 2b) Invasive mechanical ventilation/ECMO) and 2c) death.


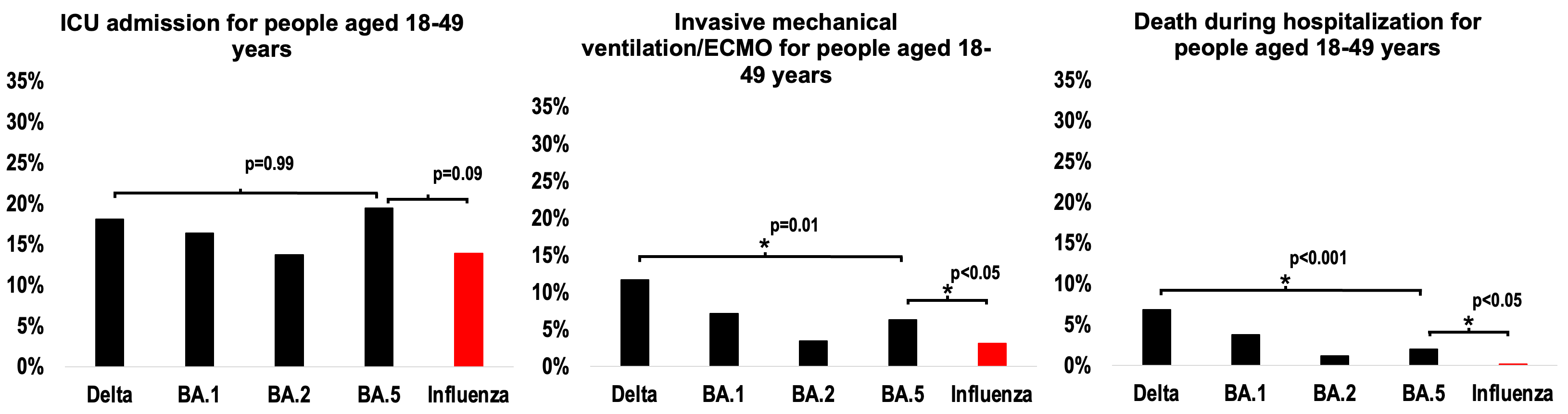
 *A statistically significant change in trend was assessed with Cochran-Armitage test; A statistically significant difference between COVID-19 Omicron BA.5 and influenza was assessed using logistic regression.

Supplemental Figure 3 (Left to right). Comparison of percentage of adult patients aged 50-64 years admitted for COVID-19 by COVID-19 variant/subvariant predominance period and a comparison of the percentage of patients admitted for COVID-19 Omicron BA.5 versus influenza, RESP-NET, 2021-2022, for the following outcomes: 3a) ICU admission 3b) Invasive mechanical ventilation/ECMO) and 3c) death.


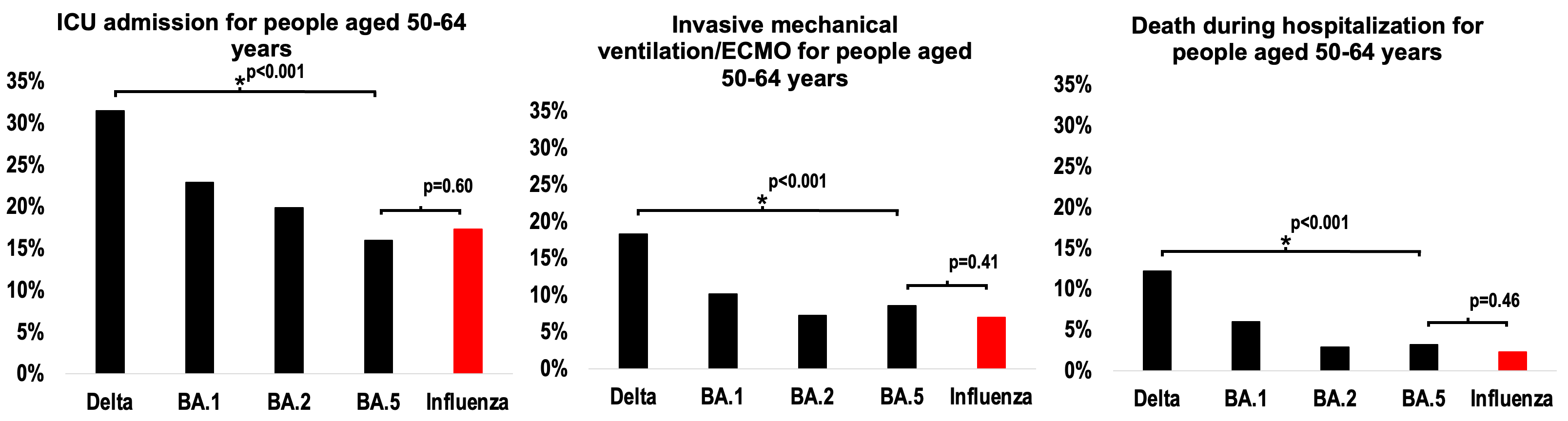
 *A statistically significant change in trend was assessed with Cochran-Armitage test; A statistically significant difference between COVID-19 Omicron BA.5 and influenza was assessed using logistic regression.

Supplemental Figure 4 (Left to right). Comparison of percentage of adult patients aged 65-74 years admitted for COVID-19 by COVID-19 variant/subvariant predominance period and a comparison of the percentage of patients admitted for COVID-19 Omicron BA.5 versus influenza, RESP-NET, 2021-2022, for the following outcomes: 4a) ICU admission 4b) Invasive mechanical ventilation/ECMO) and 4c) death.


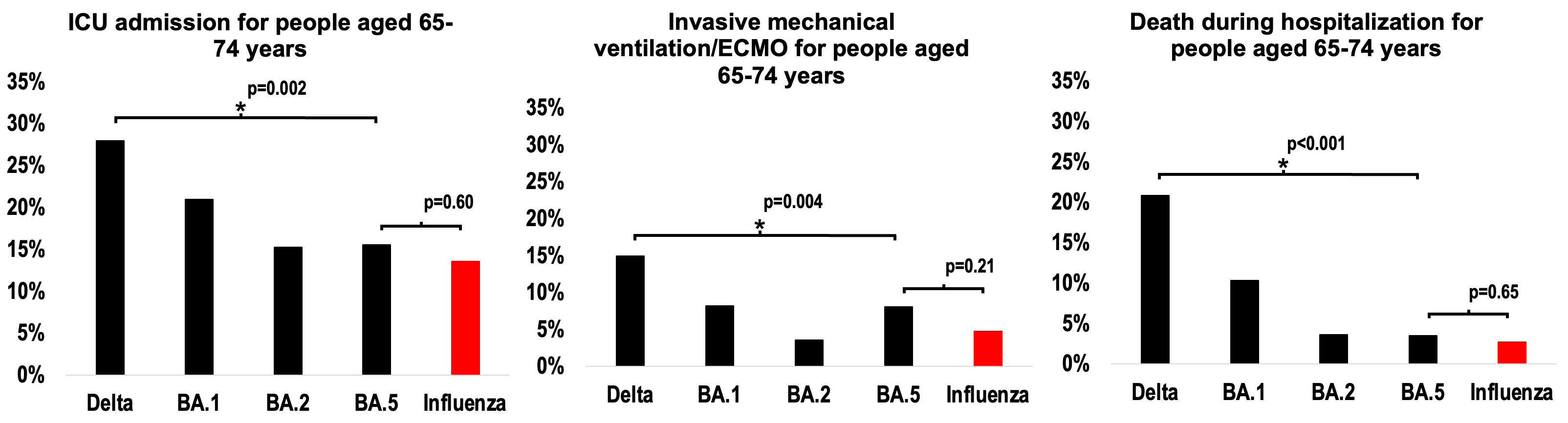
 *A statistically significant change in trend was assessed with Cochran-Armitage test; A statistically significant difference between COVID-19 Omicron BA.5 and influenza was assessed using logistic regression.

Supplemental Figure 5 (Left to right). Comparison of percentage of adult patients aged ≥75 years admitted for COVID-19 by COVID-19 variant/subvariant predominance period and a comparison of the percentage of patients admitted for COVID-19 Omicron BA.5 versus influenza, RESP-NET, 2021-2022, for the following outcomes: 5a) ICU admission 5b) Invasive mechanical ventilation/ECMO) and 5c) death.


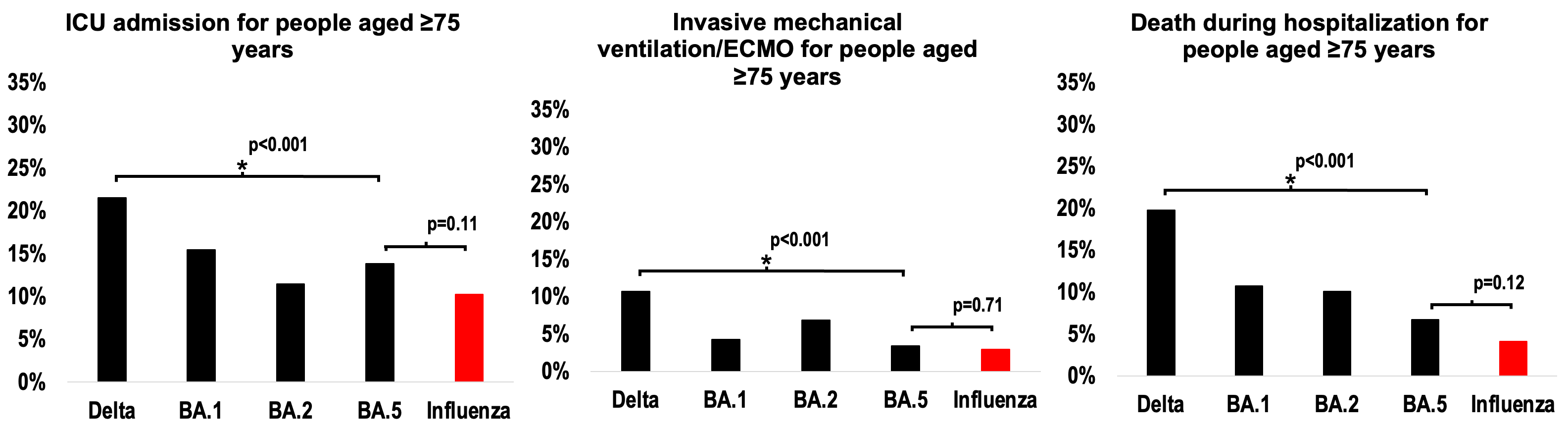
 *A statistically significant change in trend was assessed with Cochran-Armitage test; A statistically significant difference between COVID-19 Omicron BA.5 and influenza was assessed using logistic regression.
